# Alcohol use disorders in the emergency ward: choice of the best mode of assessment and identification of at-risk situations

**DOI:** 10.1186/1865-1380-4-27

**Published:** 2011-06-14

**Authors:** Charlotte Richoux, Isabelle Ferrand, Enrique Casalino, Benoit Fleury, Christine Ginsburg, Michel Lejoyeux

**Affiliations:** 1 Hopital Maison Blanche, Paris, France; 2 Department of Psychiatry. Hopital Bichat. AP-HP Paris, France; 3 Department of Psychiatry. Hopital Cochin. AP-HP. Paris, France; 4 Emergency ward. Hopital Bichat. AP-HP. Paris, France; 5 Emergency ward. Centre hospitalier universitaire. Bordeaux, France; 6 Emergency ward. Hopital Cochin. AP-HP. Paris, France

## Abstract

**Background:**

This study aims to identify the prevalence and at-risk situations of alcohol use disorders among patients examined in the emergency department and to compare the scales commonly used to identify alcohol use disorders.

**Methods:**

We used the CAGE and AUDIT questionnaires and a structured interview, the MINI.

**Findings:**

Of the presenting patients, 9.5% met the DSM-IV criteria for alcohol use disorders. The CAGE questionnaire was less sensitive (75%) and more specific (92%) than the AUDIT (87 and 80%, respectively). The typical alcohol-dependent patient is a young man who is unemployed and brought to the emergency department by the police. During the past 24 h, he has consumed alcohol, nicotine, cocaine, sedatives or cannabis.

**Conclusion:**

Of the patients, 9.5% examined in the emergency department present with alcohol abuse or dependence without asking spontaneously for treatment for their addiction. These results support the importance of systematically identifying alcohol use disorders with a simple and rapid questionnaire such as the CAGE questionnaire.

## Introduction

Alcohol use disorders are one of the most frequent diagnoses among patients examined in an emergency ward [1]. D'onofrio et al. noticed that 25% of the people admitted to an emergency ward showed misuse of alcohol [2]. Despite this high frequency rate, alcohol use disorders are usually not diagnosed in the emergency ward [2], and patients do not ask for specific help related to alcohol.

## Aim of the study

### Our work had two objectives

- To compare the acceptability and utility of two commonly used clinical scales, the CAGE [3] and AUDIT (Alcohol Use Disorders Identification Test) [4], to a structured clinical interview, the Mini International Neuropsychiatric Interview (MINI) [5], checking the DSM-IV-TR (Diagnostic and Statistical Manual of Mental Disorders, Fourth Edition revised) [6] criteria for alcohol abuse and dependence,

- To identify at-risk situations for alcohol use disorders.

## Methods

All participants aged 18 years and over presented to one of the three French emergency wards participating in the study (Bichat, Cochin-Paris and Saint-André-Bordeaux). Each year Bichat hospital receives 80,000 medical, surgical and psychiatric emergency patients from the northern districts of Paris. Cochin Hospital receives 47,000 emergency patients each year from the center of Paris and Bordeaux Hospital 22,000 each year.

Over a 6-month period (September 2008 to March 2009), we interviewed 1,079 consecutive patients, equally divided among the three wards. Interviews were conducted between 9 a.m. and 7 p.m. by the same investigator (C.R.). We proposed the assessment to all consecutive patients presenting to the emergency ward during the study period. We did pre-select the patients. Because of the severity of their somatic states (extreme pain, shell-shocked, needing intensive reanimation), 100 patients who couldn't answer the questionnaire were not included. Among the patients not included, 15 were unable to participate because of too high alcohol consumption. All other patients in a state of inebriation were assessed when their alcohol level decreased and when they were sufficiently alert to understand the questionnaire. Globally, 1% of patients were not included, and 0.1% (19) refused to answer the questionnaire.

Our work was presented to and approved by the ethics committee of our department. All patients participated voluntarily in the study. To ensure confidentiality, all identifying data were removed, and records were kept under lock and key. Written informed consent was obtained.

### Sociological and demographic characteristics, medical history and psychiatric status

We collected demographic details (sex, age, work and family status) with a specific, previously validated questionnaire [7]. We noted the mode of access to the emergency department, the type of emergency, and the number of consultations and hospitalizations during the last year.

### Alcohol, nicotine and cannabis consumption, abuse and dependence, behavioral addiction

We checked the DSM-IV-TR diagnostic criteria for alcohol abuse and dependence with a specific section of the Mini International Neuropsychiatric Interview (MINI). We also used the AUDIT scale and the CAGE Questionnaire. AUDIT is a 10-item questionnaire used to screen for alcohol misuse disorder. A score of 8 or more indicates an alcohol use disorder. The CAGE is a 4-item screening tool. A score of 2 or more indicates an alcohol use disorder.

## Questions on the CAGE questionnaire

**C **Have you ever felt you should *cut *down on your drinking?

**A **Have people *annoyed *you by criticizing your drinking?

**G **Have you ever felt bad or *guilty *about your drinking?

**E **Have you ever had a drink first thing in the morning to steady your nerves or get rid of a hangover (*eye*-opener)?

## AUDIT Questionnaire

**1**. How often do you have a drink containing alcohol?

Never

Monthly or less

2-4 times a month

2-3 times a week

4 or more times a week

**2**. How many standard drinks containing alcohol do you have on a typical day when drinking?

1 or 2

3 or 4

5 or 6

7 to 9

10 or more

**3**. How often do you have six or more drinks on one occasion?

Never

Less than monthly

Monthly

Weekly

Daily or almost daily

**4**. During the past year, how often have you found that you were not able to stop drinking once you had started?

Never

Less than monthly

Monthly

Weekly

Daily or almost daily

**5**. During the past year, how often have you failed to do what was normally expected of you because of drinking?

Never

Less than monthly

Monthly

Weekly

Daily or almost daily

**6**. During the past year, how often have you needed a drink in the morning to get yourself going after a heavy drinking session?

Never

Less than monthly

Monthly

Weekly

Daily or almost daily

**7**. During the past year, how often have you had a feeling of guilt or remorse after drinking?

Never

Less than monthly

Monthly

Weekly

Daily or almost daily

**8**. During the past year, have you been unable to remember what happened the night before because you had been drinking?

Never

Less than monthly

Monthly

Weekly

Daily or almost daily

**9**. Have you or someone else been injured as a result of your drinking?

No

Yes, but not in the past year

Yes, during the past year

**10**. Has a relative or friend, doctor or other health worker been concerned about your drinking or suggested you cut down?

No

Yes, but not in the past year

Yes, during the past year

We studied the sensitivity and specificity of these two scales by comparing the number of patients that they identified as presenting an alcohol disorder. Using a standardized questionnaire [7], we quantified the number of drinks taken each day. A drink was defined as the amount of alcohol (10 g) found in 300 ml of beer, 100 ml of wine or 25 ml of whisky. Cigarette smoking was studied with the Fagerström questionnaire [8] and the DSM-IV-TR criteria for nicotine dependence. Lastly, we checked the DSM-IV-TR criteria for nicotine, opiates, cocaine, sedatives and cannabis abuse and dependence [9].

## Statistical Analysis

### Statistical methods

We used Student's *t*-test to compare continuous variables. For categorical data, we studied differences between proportions with the multiple χ^2^-test. Statistical significance was determined at the 0.05 level of confidence.

## Results

### Comparison of acceptability, sensitivity and specificity of the CAGE and AUDIT questionnaires

The standardized MINI interview showed that 9.5% of the patients (*N *= 103) presented misuse with alcohol abuse or dependence disorders. The CAGE questionnaire identified alcohol problems in 6.1% of the 1,079 patients examined in the emergency department, and the AUDIT scale showed a prevalence rate for alcohol use disorders of 9.4%. Considering the DSM-IV-R criteria diagnosis as a reference, we found that the CAGE questionnaire identified 77 true-positive and 897 true-negative cases, and the AUDIT 90 true-positive and 780 true-negative cases. The sensitivity (true-positive cases) of the CAGE questionnaire was 75% (77/103). The AUDIT sensitivity rate was higher at 87% (90/103). The CAGE questionnaire had a higher rate of specificity [true-negative cases; 92% (897/976)] than the AUDIT [80% (780/976)].

### Identification of patients at-risk for alcohol use disorders

We compared patients identified as alcohol abusers or dependent with the MINI questionnaire and with the DSM-IV-TR criteria (Alcohol + group) to those who did not fulfill these criteria (Alcohol - group). Forty-one percent had been previously treated for alcohol use disorders, and 23% hospitalized for a reason directly related to alcohol abuse or dependence. Patients from the Alcohol + group were younger, and the ratio of men was higher. They were more often unemployed, had taken more days of sick leave during the last year (3.3 versus 1.6 days, respectively) and were more often brought to the emergency department by police. Alcohol + patients drank larger amounts of alcohol and presented more often with acute intoxication. They smoked more cigarettes and more often demonstrated cannabis, opiate, cocaine and sedative dependence (tables 1 and 2).

**Table 1:** Socio-demographic and addictive characteristics of patients examined in an emergency ward presenting with and without alcohol use disorders

	Alcohol use disorders *N *= 103	Non-alcohol use disorders *N *= 976	All patients *N *= 1,079	Statistics
Age: mean (SD)	42.7 (15)	47 (20)	46.6 (20)	Student's *t *= -2
Range	18-74	18-94	18-94	df = 1,077, *p *= 0.01
Gender				
Men	61 (59%)	432 (45%)	493 (46%)	χ^2 ^= 8.4, df = 1, *p *= 0.004
Women	42 (41%)	544 (55%)	586 (54%)	χ^2 ^= 8.4, df = 1, *p *= 0.004
Work status				
Working	53 (51%)	563 (58%)	616 (57%)	χ^2 ^= 1.4, df = 1, *p *= 0.22
Unemployed	13 (13%)	63 (6%)	76 (7%)	χ^2 ^= 5.4, df = 1, *p *= 0.02
Days of sick leave mean (SD) in the year	3.3 (3.9)	1.6 (20.6)	3.2 (19)	Student's *t *= -2,df = 1,077, *p *= 0.38
Family				
Lives alone	57 (55%)	560 (57%)	617 (55%)	χ^2 ^= 0.1, df = 1, *p *= 0.7
Lives in family	46 (44%)	416 (42%)	462 (42%)	χ^2 ^= 0.1, df = 1, *p *= 0.7
Consumption of psycho-active agents during the day of assessment in the emergency ward				
Alcohol	64 (62%)	93 (9.5%)	157 (14.5%)	χ^2 ^= 203, df = 1, *p *< 0.0001
Nicotine	73 (70%)	270 (27%)	343 (31%)	χ^2 ^= 78, df = 1, *p *< 0.0001
Cannabis	10 (10%)	19 (2%)	29 (3%)	χ^2 ^= 21.4, df = 1, *p *< 0.0001
Other drugs	8 (8%)	1 (0.1%)	9 (1%)	χ^2 ^= 66, df = 1, *p *< 0.0001
Alcoholic drinks				
Number of drinks				
during weekdays Mean (SD)	19.3 (18)	1.6 (3.5)	3.3 (2.5)	Student's *t *= 25.5, df = 1,077, *p *< 0.0001
Drinks on weekend Mean (SD)	11.9 (11)	1.3 (8)	2.3 (2.2)	Student's *t *= 23.6, df = 1077, *p *< 0.0001
Drinks all week Mean (SD) 17.8 (20)	1.5 (3)	3.1 (3)	Student's *t *= 21.9, df = 1,077, *p *< 0.0001	

**Table 2:** Addictive characteristics of patients examined in an emergency ward presenting with and without alcohol use disorders

	Alcohol use disorders *N *= 103	Non-alcohol use disorders *N *= 976	All patients *N *= 1,079	Statistics
Acute alcohol intoxication				
In the month Mean (SD)	11.8 (12)	0.2 (0.6)	1.3 (3.8)	Student's *t *= 28, df = 1,077, *p *< 0.0001
History of treatment for alcohol use disorders	41 (39%)	30 (3%)	71 (6%)	χ^2 ^= 198, df = 1, *p *< 0.0001
History of group therapy	19 (19%)	13 (1%)	32 (3%)	χ^2 ^= 94, df = 1, *p *< 0.0001
Consultation with a general practitioner related to alcohol	42 (41%)	22 (2%)	64 (6%)	χ^2 ^= 247, df = 1, *p *< 0.0001
Consultation with a specialist related to alcohol	39 (38%)	27 (3%)	66 (6%)	χ^2 ^= 199, df = 1, *p *< 0.0001
Hospitalization for alcohol dependence 24 (23%)	9 (1%)	33 (3%)	χ^2 ^= 157, df = 1, *p *< 0.0001	
Nicotine use disorders				
Cigarettes/day Mean (SD)	13.4 (11)	5 (8)	5.8 (9)	Student's *t *= 8.3, df = 1,077, *p *< 0.0001
Fagerström score Mean (SD)	3.6 (3)	1.3 (2.4)	1.5 (2.6)	Student's *t *= 8.9, df = 1,077, *p *< 0.0001
Cannabis use disorders				
Cannabis abuse	11 (10%)	3 (0.3%)	14 (1.3%)	
Cannabis dependence	6 (6%)	4 (0.4%)	10 (1%)	χ^2 ^= 109, df = 2, *p *< 0.0001
Opiates use disorders				
Opiate abuse	3 (2.9%)	0	3 (0.3%)	
Opiate dependence	1 (1%)	0	1 (0.1%)	χ^2 ^= 38, df = 2, *p *< 0.0001
Cocaine use disorders				
Cocaine abuse	8 (7.7%)	1 (0.1%)	9 (0.8%)	
Cocaine dependence	1 (1%)	1 (0.1%)	2 (0.2%)	χ^2 ^= 70, df = 2, *p *< 0.0001
Sedative use disorders				
Sedative abuse	9 (9%)	7 (0.7%)	16 (1.5%)	
Sedative dependence	8 (8%)	0	8 (0.7%)	χ^2 ^= 118, df = 2, *p *< 0.0001

## Discussion

### Comparison of modes of assessment

The AUDIT questionnaire was more sensitive than the CAGE, which however allowed the identification of 75% of patients with alcohol use disorders. When the CAGE questionnaire is given without explanation by a nurse or a doctor in another context, answers to its four questions may be less accurate. The CAGE questionnaire is thus recommended to be delivered by a nurse or a doctor specifically competent in the recognition of addiction. However, the AUDIT questionnaire is still the more sensitive instrument (92% versus 75%), even if it takes more time to complete.

### At-risk situations for alcohol abuse or dependence among patients examined in the emergency department

The typical patient presenting to the emergency department with an alcohol use disorder is a young, unemployed man, usually brought to the emergency department by the police or examined after a state of agitation. During the past 24 h, he has consumed alcohol, nicotine or cannabis. He has more often been treated for alcohol abuse or dependence by a general practitioner or a specialist. In one case of four, he has been hospitalized for alcohol use disorders, and he has had more days of sick leave during the last year. He presents dependence or abuse of nicotine, cannabis, opiates, cocaine or sedatives more often.

### Consequences of the results for clinical practice and public health

None of the patients identified as an abuser of or dependent on alcohol had asked for specific help for addiction before being diagnosed by our systematic intervention. If they had not been assessed, they would have left the emergency ward with no treatment or information about the possibilities of help. The stay in the emergency ward represents the only opportunity to receive information and begin to benefit from adequate treatment.

### Limitations of the study

One of the principal limitations of our study is the exclusion of 15 patients unable to answer the questionnaire because of their high level of alcohol intoxication. Another limitation is that our results were only drawn from patients presenting to the emergency ward between 9 a.m. and 9 p.m. Our work is only based on clinical assessment without biological confirmation. In spite of these limitations, our study was the first to include such a high number of patients examined in three French emergency wards and to assess simultaneously all types of dependence.

## Conclusion

Of the patients examined in the emergency department, 9.5% met the DSM-IV-R criteria of alcohol use disorders. In the context of an emergency, the CAGE questionnaire was less sensitive (75% versus 87%) than the AUDIT scale and more specific (92% versus 87%). None of the patients identified as alcohol abusers or dependent made a spontaneous request for treatment. Patients with alcohol disorders were more often men, younger, referred to the emergency ward after a crisis and agitation, and were brought in by the police. They presented a multi-addictive disorder associating nicotine, cannabis, opiate and sedative dependence disorders. These data support the importance of systematically identifying alcohol use disorders in the emergency department and initiating treatment for these patients who would have remained unknown without a specific assessment.

## Competing interests

The authors declare that they have no competing interests.

## Authors' contributions

ML, IF and CR conceived the study. CR assessed all patients. EC assessed patients in BH, BF in Bordeaux hospital and CG in Cochin Hospital. All authors read and approved the final manuscript.
